# Wrist accelerometer shape feature derivation methods for assessing activities of daily living

**DOI:** 10.1186/s12911-018-0671-1

**Published:** 2018-12-12

**Authors:** Matin Kheirkhahan, Avirup Chakraborty, Amal A. Wanigatunga, Duane B. Corbett, Todd M. Manini, Sanjay Ranka

**Affiliations:** 1 Department of Computer and Information Science and Engineering, University of Florida, Gainesville, FL USA; 2 Department of Epidemiology, Johns Hopkins University, Baltimore, MD USA; 3 Department of Aging and Geriatric Research, University of Florida, Gainesville, FL USA

**Keywords:** Physical activity, Big data, ActiGraph, Energy expenditure, Bag of words

## Abstract

**Background:**

There has been an increasing interest in understanding the usefulness of wrist-based accelerometer data for physical activity (PA) assessment due to the ease of use and higher user compliance than other body placements. PA assessment studies have relied on machine learning methods which take accelerometer data in forms of variables, or feature vectors.

**Methods:**

In this work, we introduce automated shape feature derivation methods to transform epochs of accelerometer data into feature vectors. As the first step, recurring patterns in the collected data are identified and placed in a codebook. Similarities between epochs of accelerometer data and codebook’s patterns are the basis of feature calculations. In this paper, we demonstrate supervised and unsupervised approaches to learn codebooks. We evaluated these methods and compared them with the standard statistical measures for PA assessment. The experiments were performed on 146 participants who wore an ActiGraph GT3X+ accelerometer on the right wrist and performed 33 activities of daily living.

**Results:**

Our evaluations show that the shape feature derivation methods were able to perform comparably with the standard wrist model (F1-score: 0.89) for identifying sedentary PAs (F1-scores of 0.86 and 0.85 for supervised and unsupervised methods, respectively). This was also observed for identifying locomotion activities (F1-scores: 0.87, 0.83, and 0.81 for the standard wrist, supervised, unsupervised models, respectively). All the wrist models were able to estimate energy expenditure required for PAs with low error (rMSE: 0.90, 0.93, and 0.90 for the standard wrist, supervised, and unsupervised models, respectively).

**Conclusion:**

The automated shape feature derivation methods offer insights into the performed activities by providing a summary of repeating patterns in the accelerometer data. Furthermore, they could be used as efficient alternatives (or additions) for manually engineered features, especially important for cases where the latter fail to provide sufficient information to machine learning methods for PA assessment.

## Background

Physical activity (PA) assessment has been widely pursued in a variety of research studies including determining relationship with health status [1], detecting future hazardous events (e.g., major mobility disability) [2], and evaluating the performance of interventional studies aimed to increase PA [3]. For PA assessment, wearable accelerometers have been the main devices for data collection, which are minimally intrusive and provide objectively accurate measurements. Most of the studies have been conducted with the accelerometer attached to the hip, since the information obtained from this body placement was highly correlated with ambulation [4]. There has been a recent interest in understanding the usefulness of wrist-based accelerometer data collection due to ease of use and higher compliance. In particular, smartwatches play an important role in this change, since they are popular and have necessary resources, e.g., sensor monitors and connectivity means, to make real-time mobility monitoring possible. There have been several remote health monitoring frameworks proposed by researchers that are dependent on wrist-accelerometer PA assessment models [5].

PA assessment approaches include two main parts: 1) PA identification, which is a classification task and 2) energy expenditure estimation for performing the PA, which is a regression problem. In both areas, it has been shown that machine learning methods perform better than statistical regression-based approaches [6]. There are two most widely used approaches to define a physical activity in the PA assessment studies. While some works focused on recognizing homogeneous activities, such as sitting, standing, and walking [7], there have been other works that attempted to detect activities of daily living, e.g., grocery shopping and yard work, which are composites of homogeneous activities [8]. Each approach has unique advantages; however, the latter definition of physical activity has been more closely investigated to assess lifestyles of individuals [6].

Most machine learning methods require data in the form feature vectors, and therefore, their performance depends on the feature derivations. Statistical summaries of accelerometer data have been the most popular choice for feature derivation in PA assessment studies. This type of feature derivation is a manual procedure, which requires domain expertise and sufficient knowledge about the relation between the data in hand and the target variable. To assess activities of daily using wrist accelerometer data, a set of seven summary statistics for short epochs (12.8 or 15 s) has been introduced [9]. The feature vectors obtained from this approach has been shown to be effective measurements for detecting sedentary and locomotion activities [6, 10].

When summarizing a dataset into a few summary statistics, it is possible that two different activities exhibit similar features. In this method, we lose certain details and information that might be useful to identify other types of activities. Therefore, for a new target variable (e.g., a new activity of interest), another set of features might be required. The present work, which builds on a previous paper presented by Kheirkhahan et al. [11], proposes two shape feature derivation methods for PA assessment that are based on detecting representative accelerometer patterns in a dataset containing activities of daily living. The representative patterns are called *atoms* and gathered in a *codebook*. The learned codebook is used to calculate feature vectors based on similarities of subsequences within a PA accelerometer data and atoms.

The main contributions of the present work are:

- We introduce an unsupervised method to learn a comprehensive codebook from the wrist accelerometer data. We obtain a bag-of-words representation of the data by replacing the subsequences of data with their most similar atoms. We apply a term frequency-inverse document frequency function on bag-of-words representations to obtain feature vectors suitable to be passed to PA assessment models.
- Leveraging the idea of motif detection [12], we propose a supervised shape feature derivation method that identifies recurring patterns for an activity of interest. We show that this method results in a more specialized codebook with fewer atoms, and therefore, the distance of subsequences of data to atoms are directly used as new features.
- We evaluate the three different feature derivation methods for PA assessment, which are 1) standard wrist model relying on statistical summaries, 2) bag-of-words approach, and 3) supervised shape feature. The present work shows that the shape features perform with high accuracy for identifying sedentary and locomotion PAs. There accuracies are comparable with the statistical features that were defined for this problem. However, they provide further information that contribute to models with higher accuracy than the standard wrist model for new activities of interest.
- The current study also investigates whether performing additional steps of activity identification improve the energy expenditure estimation for all three models.

## Methods

### Data Collection

#### Participants

One hundred and forty six adults with ages ranging between 20 and 89 years participated in a study of metabolic costs of daily activity [13]. Table 1 shows the participant characteristics. The participants were community dwelling adults, who were required to understand and speak English, had stable body weight for at least three months, and willing to undergo all testing procedures. They provided written informed consents approved by the Institutional Review Board at the University of Florida.

**Table 1:** Participant characteristics

Characteristic	All (*n*=146)	Women (*n*=97)	Men (*n*=49)
Age (years)	58.6±17.4	59.2±17.0	57.6±18.2
Weight (kg)	74.8±17.1	68.9±14.6	86.3±15.7
Height (m)	167.5±9.2	163.0±6.2	176.4±7.7
BMI (kg/m^2^)	25.9±6.5	25.3±6.7	27.1±5.7

BMI, body mass index

They were asked to perform a list of 33 tasks mimicking daily chores in four visits, including exercise and sedentary type physical activities, in a clinic laboratory setting (see Table 2). An ActiGraph GT3X+ (ActiGraph, Pensacola, FL) triaxial accelerometer was mounted on their right wrist. Locomotion tasks were performed during the first visit, while the fourth visit was dedicated to sedentary tasks (e.g., TV watching and standing still). During the other two visits, participants engaged in tasks where ambulation was necessary for some parts. For computer work, TV watching, and strength exercises, participants were asked to remain seated throughout the activity.

**Table 2:** Summary statistics for the activities of daily living performed in the laboratory setting

Activity	Locomotion	Sedentary	METs	MVM	SDVM	MANGLE	SDANGLE	P625	DF	FPDF
Stair Ascent	Yes	No	6.36 (1.93)	1.07 (0.03)	0.17 (0.05)	-19.88 (22.72)	21.52 (5.54)	0.52 (0.07)	1.47 (0.51)	0.07 (0.02)
Walking at RPE 5	Yes	No	4.85 (1.19)	1.25 (0.18)	0.25 (0.08)	-41.26 (41.90)	12.92 (3.85)	0.56 (0.09)	1.59 (0.53)	0.14 (0.03)
Rapid Walk	Yes	No	4.64 (1.42)	1.24 (0.15)	0.26 (0.09)	-41.48 (42.75)	12.99 (4.45)	0.52 (0.13)	1.73 (0.70)	0.12 (0.03)
Heavy Lifting	No	No	4.43 (0.92)	1.06 (0.02)	0.15 (0.03)	-26.30 (22.71)	24.26 (3.95)	0.52 (0.03)	1.45 (0.26)	0.07 (0.01)
Yard Work	No	No	4.33 (1.15)	1.08 (0.03)	0.21 (0.05)	-23.37 (24.60)	22.25 (4.26)	0.52 (0.05)	1.20 (0.54)	0.06 (0.01)
Digging	No	No	4.22 (1.24)	1.07 (0.04)	0.24 (0.11)	-27.29 (29.77)	25.30 (7.85)	0.50 (0.05)	1.43 (0.63)	0.05 (0.01)
Trash Removal	No	No	3.90 (0.74)	1.07 (0.02)	0.19 (0.04)	-25.75 (22.56)	23.24 (3.07)	0.54 (0.04)	1.40 (0.27)	0.07 (0.01)
Vacuuming	No	No	3.73 (0.68)	1.06 (0.04)	0.12 (0.03)	-32.06 (28.55)	16.06 (3.32)	0.51 (0.03)	1.25 (0.28)	0.06 (0.01)
Mopping	No	No	3.55 (0.81)	1.07 (0.04)	0.19 (0.05)	-20.41 (23.29)	18.90 (5.17)	0.57 (0.06)	1.17 (0.43)	0.08 (0.02)
Replacing Sheets On a Bed	No	No	3.50 (0.72)	1.11 (0.04)	0.28 (0.06)	-20.60 (17.18)	24.22 (3.22)	0.52 (0.03)	1.24 (0.19)	0.06 (0.00)
Straightening Up and Dusting	No	No	3.42 (0.86)	1.09 (0.04)	0.17 (0.05)	-18.12 (19.84)	19.44 (3.26)	0.52 (0.05)	1.22 (0.31)	0.07 (0.01)
Stair Descent	Yes	No	3.32 (0.59)	1.07 (0.03)	0.21 (0.07)	-34.99 (31.96)	15.42 (4.96)	0.53 (0.06)	1.68 (0.49)	0.07 (0.02)
Light Home Maintenance	No	No	3.32 (0.65)	1.06 (0.02)	0.17 (0.04)	-11.47 (13.44)	25.37 (3.30)	0.51 (0.03)	1.33 (0.20)	0.06 (0.00)
Light Gardening	No	No	3.31 (1.09)	1.07 (0.03)	0.19 (0.06)	-24.78 (26.52)	20.54 (3.35)	0.50 (0.06)	1.50 (0.56)	0.06 (0.01)
Sweeping	No	No	3.29 (0.65)	1.05 (0.03)	0.17 (0.04)	-18.30 (23.13)	20.17 (3.94)	0.56 (0.05)	1.24 (0.35)	0.06 (0.01)
Walking at RPE 1	Yes	No	3.29 (0.63)	1.07 (0.04)	0.15 (0.04)	-42.44 (42.03)	9.04 (3.50)	0.56 (0.09)	1.58 (0.39)	0.13 (0.04)
Leisure Walk	Yes	No	3.26 (0.88)	1.08 (0.05)	0.16 (0.05)	-43.84 (42.76)	8.96 (3.95)	0.54 (0.15)	1.84 (0.77)	0.13 (0.03)
Washing Windows	No	No	3.17 (0.62)	1.12 (0.07)	0.28 (0.10)	-4.96 (11.70)	22.04 (3.26)	0.53 (0.04)	1.30 (0.24)	0.07 (0.01)
Laundry	No	No	2.85 (0.74)	1.06 (0.02)	0.20 (0.04)	-15.43 (13.47)	23.94 (2.74)	0.53 (0.03)	1.36 (0.25)	0.06 (0.00)
Prepare and Serve Meal	No	No	2.68 (0.65)	1.05 (0.02)	0.13 (0.03)	-14.65 (15.30)	16.65 (3.29)	0.47 (0.04)	1.79 (0.36)	0.06 (0.01)
Dressing	No	No	2.61 (0.54)	1.06 (0.02)	0.15 (0.03)	-13.19 (15.19)	20.98 (3.10)	0.49 (0.03)	1.57 (0.25)	0.05 (0.00)
Unloading and Storing Dishes	No	No	2.55 (0.47)	1.05 (0.03)	0.16 (0.03)	2.39 (10.28)	20.23 (3.04)	0.49 (0.04)	1.55 (0.30)	0.05 (0.01)
Shopping	No	No	2.42 (0.46)	1.04 (0.02)	0.09 (0.02)	-12.32 (13.13)	16.11 (3.18)	0.50 (0.03)	1.41 (0.26)	0.06 (0.01)
Personal Care	No	No	2.36 (0.47)	1.08 (0.04)	0.21 (0.06)	-0.94 (10.50)	27.30 (3.70)	0.50 (0.05)	1.55 (0.33)	0.06 (0.01)
Yoga	No	No	2.31 (0.62)	1.02 (0.01)	0.06 (0.03)	-32.42 (28.01)	11.29 (4.12)	0.49 (0.03)	1.37 (0.26)	0.06 (0.01)
Ironing	No	No	2.19 (0.41)	1.05 (0.02)	0.13 (0.03)	-6.17 (9.09)	16.94 (2.85)	0.50 (0.02)	1.67 (0.22)	0.05 (0.00)
Washing Dishes	No	No	2.16 (0.39)	1.06 (0.03)	0.14 (0.04)	-13.30 (14.36)	13.98 (2.45)	0.46 (0.04)	1.54 (0.34)	0.06 (0.01)
Strength Exercise										
Leg Extension	No	No	2.01 (0.63)	1.03 (0.02)	0.03 (0.02)	-12.60 (16.5)	6.04 (3.21)	0.42 (0.04)	2.00 (0.52)	0.06 (0.02)
Leg Curl	No	No	2.00 (0.73)	1.03 (0.02)	0.03 (0.02)	14.03 (17.81)	5.95 (3.46)	0.41 (0.03)	2.40 (0.50)	0.05 (0.01)
Chest Press	No	No	1.86 (0.55)	1.03 (0.02)	0.06 (0.03)	16.04 (19.26)	9.69 (4.06)	0.46 (0.05)	1.28 (0.36)	0.08 (0.01)
Computer Work	No	Yes	1.24 (0.24)	1.03 (0.02)	0.03 (0.02)	0.83 (8.25)	3.99 (2.68)	0.38 (0.04)	2.65 (0.56)	0.05 (0.01)
Standing Still	No	Yes	1.22 (0.29)	1.02 (0.01)	0.01 (0.01)	-33.35 (35.30)	3.63 (3.26)	0.42 (0.04)	1.73 (0.57)	0.09 (0.05)
TV Watching	No	Yes	1.11 (0.41)	1.03 (0.02)	0.01 (0.01)	-8.04 (18.5)	2.80 (1.67)	0.39 (0.03)	1.78 (0.62)	0.10 (0.04)
MET, metabolic equivalent score										

#### Instrumentation

The ActiGraph GT3X+ monitors were mounted on participants’ right wrists. These lightweight accelerometers record accelerations in units of gravity (1 g) in perpendicular, anterior-posterior, and medio-lateral axes. They were configured to collect data at 100 Hz sampling rate. Energy expenditures was measured in parallel using a portable indirect calorimetry system, Cosmed K4b2 [14]. Respiratory gas exchange data were collected breath-by-breath through a fitted mask. Data were converted to MET values defined as the oxygen uptake (VO_2_ = ml/min.kg) during steady state rate expressed as a function of 3.5 ml/min.kg.

### Wrist Accelerometer Models

A conventional accelerometer model for activity recognition consists of modules for feature vector calculations from the time-series data and machine learning methods for predicting activity labels. In this work, we present three different approaches for feature representations; 1) statistical summaries of accelerometer data, which has been the standard feature vectors for physical activity assessments, 2) a bag-of-words approach, which is an unsupervised method for detecting recurring patterns of accelerations in the unprocessed time-series data, and 3) a supervised shape feature derivation, which identifies epochs of acceleration data that are repeated within an activity of interest. Details for each method are presented in the following:

#### Standard Statistical Wrist Feature Representation

We develop the wrist model (i.e., set of variables) using which have been widely used for activity recognition and physical activity assessment using high-frequency accelerometer data collected on the wrist [6, 9, 15]. Briefly, the constructed variables are:

- Time-domain variables: the mean and standard deviation vector magnitude (MVM and SDVM, respectively), where the vector magnitude is calculated using *x*, *y*, and *z*-axes of the accelerometer as follows:
  $$VM = \sqrt{x^{2} + y^{2} + z^{2}}$$
- Frequency-domain variables: after obtaining the frequency representation of the vector magnitude using the Fast Fourier Transform [16], the following variables are calculated: 1) the dominant frequency (DF), 2) its fraction of power (FPDF), and 3) the fraction of power within human movement frequencies, i.e., 0.6 Hz to 2.5 Hz (P625).
- Orientation-related variables: the mean and standard deviation of the existing angle between the perpendicular axis (*x*) and vector magnitude (MANGLE and SDANGLE, respectively), where the angle is calculated as follows:
  $$\text{angle} = \frac{180}{\pi} \times sin^{-1} \frac{x}{VM}$$

We calculate the above-mentioned variables from non-overlapping 15-s epochs of acceleration data.

#### Bag-of-Words Feature Representation

**Fig. 1:**
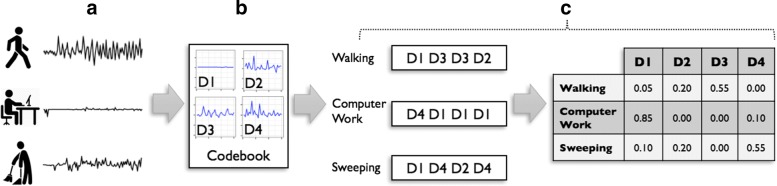
Bag-of-words variable representation. **a** First, the most common acceleration patterns (i.e., *atoms*) are discovered and gathered in a *codebook*. **b** Second, the time-series accelerometer data for a PA are split into subsequences. **c** Each subsequence is replaced with the label of the most resembling atom. After this step, the accelerometer data are converted to *bag of words*, which are used to calculate word frequencies for each PA. A normalization term-frequency function (i.e., inverse document frequency) is applied to adjust the values of the variable vectors (i.e., word frequencies) and make them suitable for machine learning methods

Every observation (i.e., accelerometer data for a PA) is considered a time series sequence. The bag-of-words time series model is a three-pronged process, which is depicted in Fig. 1. At the first step, we find *k*-second accelerometer patterns that are representative of vector magnitudes in the collected data. These representative patterns are often called *atoms* [17] and in the physical activity literature, the duration for such patterns (*k*) is usually less than 6 s [18]. Atoms are gathered into a *d*-atom *codebook*. After finding the codebook, we convert every *k*-second subsequence of accelerometer data to a *word* using the label of the atom which resembles that subsequence the most. Using the word representation of the accelerometer data, we calculate normalized word frequency variables to obtain numeric vectors suitable for the machine learning methods. Details of this method is presented in the following paragraphs.

##### Codebook learning

**Fig. 2:**
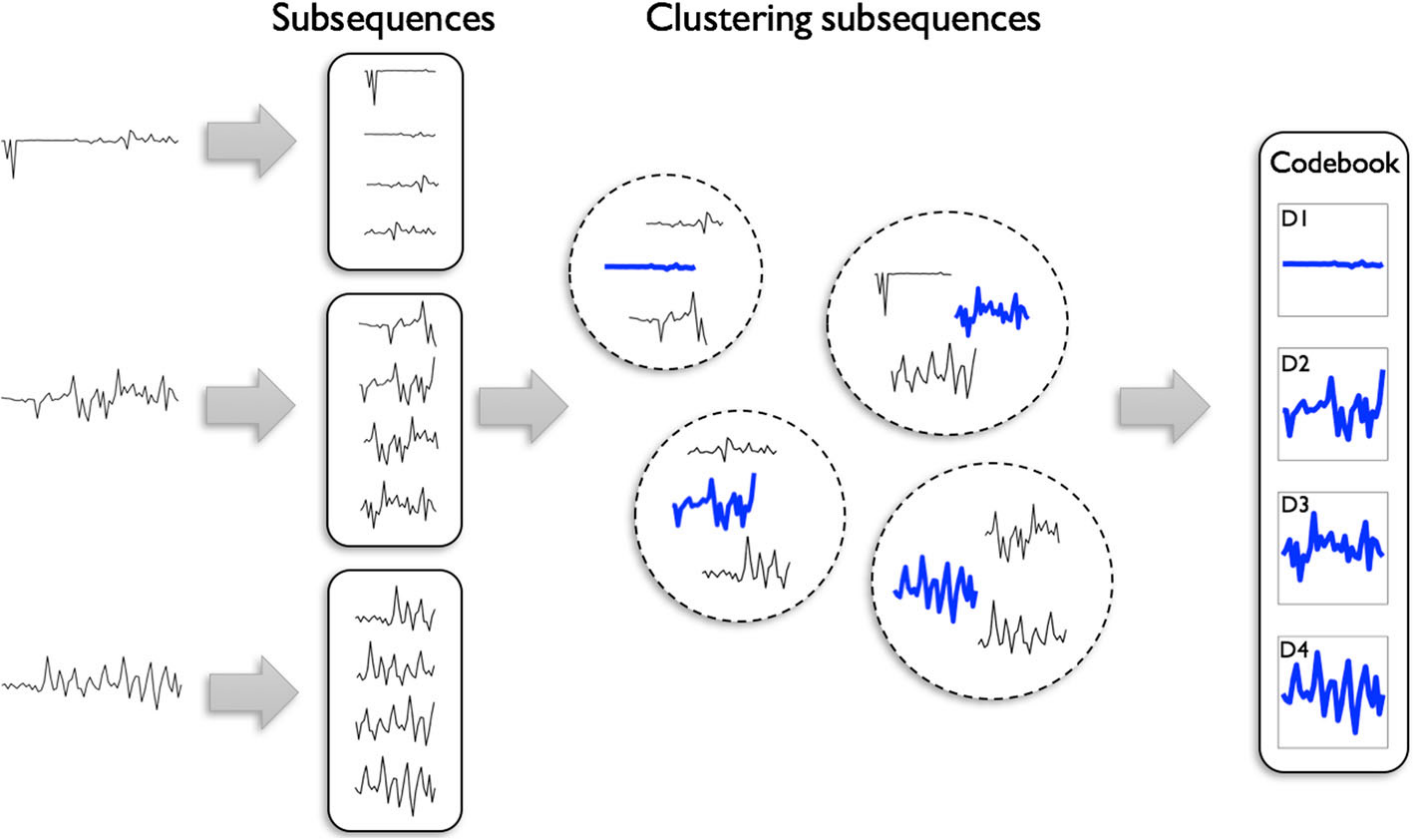
BoW codebook learning

To find a comprehensive codebook containing the representative acceleration patterns, we divide each time series data into subsequences. Subsequences are *k*-second subintervals of the original time series instance, where the two adjacent subsequences have *k*−1 s of overlapping parts. These subsequences are obtained using a *k*-second sliding window, which slides 1 s after each subsequence subtraction. For a sampling rate of *s* Hz (*s* acceleration values for every second), each subsequence has *k*^′^=*k*×*s* data points. More specifically, from the *i*^*th*^ time-series data with *t*_*i*_ s duration, we extract *t*_*i*_−*k*+1 subsequences, each having *k*^′^ data points. Therefore, for a dataset containing *n* observations we will obtain ${\sum \nolimits }_{i = 1}^{n}(t_{i} - k + 1)$ subsequences. Next, we apply clustering algorithms to obtain *d* representative acceleration patterns and learn the codebook. For a study with 150 participants, each performing thirty 10-min long PAs and the subsequence length (*k*=) 3 s, we obtain more than 2.5 billion subsequences. To be able to cluster this large number of subsequences, we use Apache Spark Big Data framework for partition-based clustering (Spark MLlib [19]). The implicit parallelism of this framework provides efficient calculations suitable for large datasets. We calculate the silhouette values for clusters to find the best cluster fits [20]. The silhouette variable for the *i*^*th*^ cluster is defined as follows:

$$sl(i) = \frac{b(i) - a(i)}{max\{a(i), b(i)\}} $$

where *a*(*i*) represents the average distance between the samples within *i*^*th*^ cluster and *b*(*i*) is the minimum average distance of *i*^*th*^ cluster’s samples to other clusters. *sl*(*i*) values close to 1 indicate clusters with homogenous and uniformly distributed samples. For a *d*-atom codebook, each cluster centroid is an atom with *k*^′^ sample points in the codebook $C \in \mathbb {R}^{k^{\prime }\times d}$. Figure 2 shows the process of learning the codebook.

##### Feature calculation

**Fig. 3:**
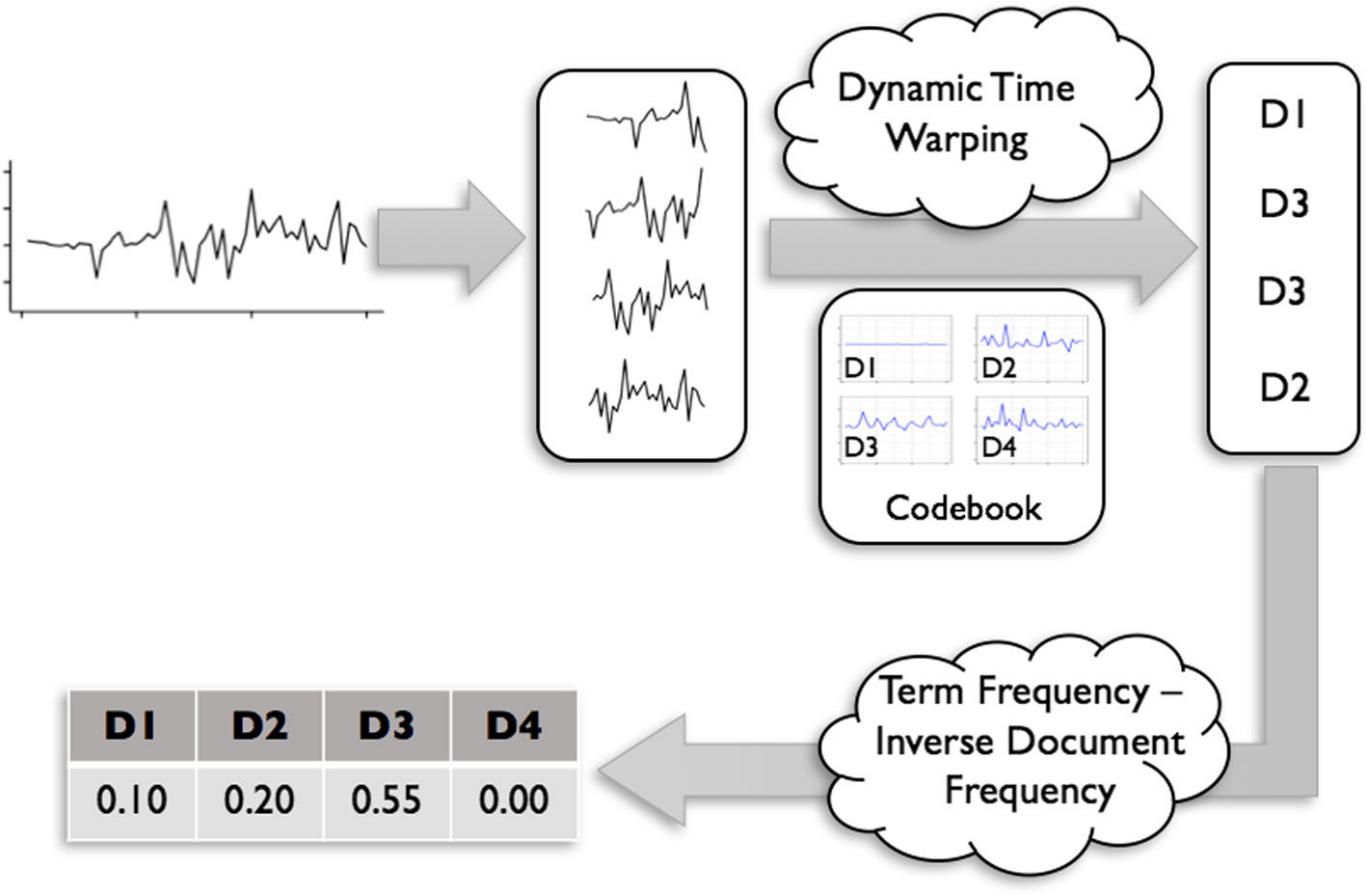
BoW variable calculation

Once the codebook is learned, we convert every time-series observation to a bag of words using the procedure depicted in Fig. 3. First, each subsequence of the time-series data is replaced by the most resembling atom in the codebook. Next, we replace each subsequence with its corresponding atom’s label to obtain the bag-of-words representation of the data. Finally, we calculate a normalized term frequency variable for the present words.

There are three major challenges in finding the most similar atom for each subsequence. First, acceleration patterns’ differences might be due to high-frequency noises, and not because of human movements. Second, the source of dissimilarity might be due to a phase delay, such that one acceleration pattern is identical to another one but shifted in time. Third, due to the difference in participants’ movements speed, an acceleration pattern obtained from one participant might be a scaled version of the other one. To address the first challenge, we apply a low-pass filter to exclude data irrelevant to human movement (i.e., >5 Hz). We also use overlapping subsequences, such that they have one second non-overlapping parts to overcome the phase-delay issue. Lastly, we employ dynamic time warping method [21] to calculate the similarity of a subsequence to codebook’s atoms. This is a dynamic programming approach which is robust to small shifts (in our case, it is <1 second) and the scaling problem.

Initially, every subsequence is represented by a word; the label of the most similar atom. Next, we construct a *d*-element vector *h*=(*h*_1_,*h*_2_,…,*h*_*d*_). Each element in this vector represents the number of subsequences which were found closest to the *i*^*th*^ atom. In the example shown in Fig. 3, we will have *h*=(1,1,2,0) using a 4-atom codebook. In our experiments, we obtain an *h* vector for every PA and calculate the term frequency (*tf*) for every word. To prevent a bias towards longer PAs, we use an augmented frequency function, which is the raw frequency of a word divided by the maximum word frequency found in the PA: $tf(i, h) = 0.5 + 0.5 \cdot \frac {h(i)}{\underset {j}{max}\{h(j)}$

Also, to detect rare and common words across all the PAs and to obtain a measure of how much information each word provides, we calculate a scaling factor, known as inverse document frequency (*idf*) as follows:

$$idf(i, H) = log \frac{n}{\sum_{h \in H}h(i)} $$

where *H* is a $\mathbb {R}^{n \times d}$ matrix containing *h* vectors for all *n* tasks. This function is effective in assigning low weights to the common words. For every PA performed by a participant, we will have *d* variables, which are calculated by multiplying the values obtained from term frequency and inverse document frequency functions. For the *i*^*th*^ word, we obtain a normalized word frequency by multiplying the abovementioned frequency terms, i.e., *tfidf*(*i*,*h*,*H*)=*tf*(*i*,*h*)·*idf*(*i*,*H*).

#### Supervised Shape Feature Representation

Similar to the bag-of-words approach, this method is also seeking to identify recurring patterns in the raw accelerometer data to form a codebook. To achieve that, this method relies on detecting *motifs* [12] that are observed within an activity of interest. This method has two major steps. First, for each participant and activity, an *initial codebook* is learned. Next, for every activity, the initial codebooks are combined and unique acceleration patterns are identified to form the final codebook.


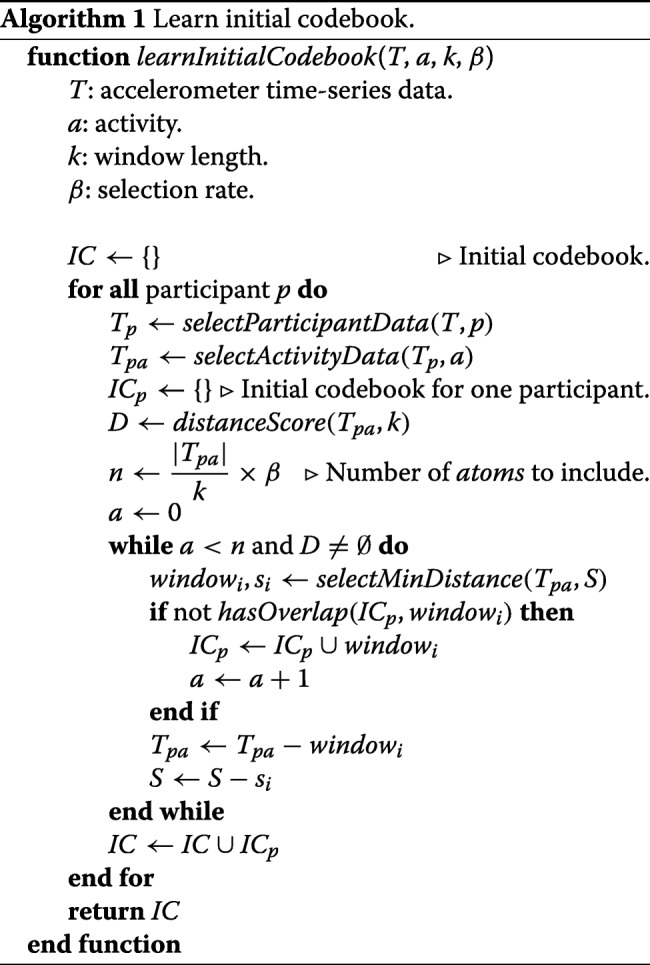


**Fig. 4:**
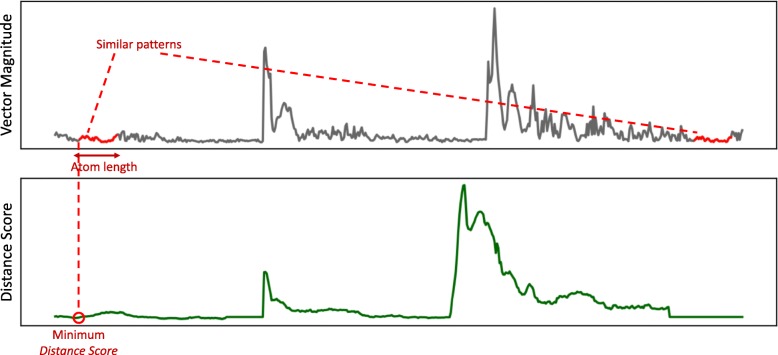
Selecting subsequence of accelerometer data (vector magnitude) with minimum distance score

The first step in this approach is to learn an initial codebook for each participant and activity and is outlined in Algorithm 1. The function takes four input arguments; 1) accelerometer time-series data (denoted as *T*) that is the vector magnitude calculated from the triaxial data of all participants, 2) the activity of interest, 3) desired length of acceleration patterns to be mined, and 4) the selection rate, which indicates what portion of data (for a participant and activity) should be considered. The algorithm calculates a vector of *distance score* (denoted as *D*) for every *k*-second subsequence of accelerometer data. To calculate the distance score, we use a sliding window approach where the distance between the selected subsequence and successor subsequences are calculated and the choose the minimum distance. Next, we include the subsequence with the minimum distance score, as an *atom*, in the *initial codebook* (denoted as *IC*). Figure 4 shows selecting a subsequence of data with the lowest distance score. The selected atom is checked to have no overlapping acceleration patterns with the previously selected atoms and after every atom selection, the corresponding subsequences are removed from the selection pool. The process of selecting atoms is repeated until we obtain the desired number of atoms for one participant. If *β* is set to 1, the initial codebook becomes similar to the pool of data chunks in the bag-of-words approach since we would include all subsequences.

When generating the initial codebook, data for each participant are considered individually since every participant performs activities with unique dominant accelerometer patterns; i.e., participants perform activities with different pace and strength. Furthermore, this allows calculating the distance scores, which has a runtime order of *O*(*n*^2^), to be expedited by parallel execution.

To learn the final codebook, we apply a hierarchical clustering method, which uses dynamic time warping as the distance function, to find groups of similar atoms. From each group, we select the medoid as the representative and include that in the final codebook, which is similar to the codebook learning step in the bag-of-words approach. To calculate a feature vector for an epoch, we use a sliding window (with 50% overlap) to calculate distances between epoch’s subsequences and atoms. The distances are calculated using the Dynamic Time Warping method. The average value of distances to each atom is used as features calculated for the epoch.

### Physical Activity Assessment

Using the derived features explained earlier, we seek to identify activities of daily living for different categories. First, we define two classification problems: 1) detecting sedentary PAs and 2) identifying locomotion activities. Sedentary PAs are defined as the activities that require <1.6 METs [22]. In our dataset, this category contains computer work, TV watching, and standing still activities. Locomotion PAs include walks, stair ascent, and stair descent. For both classification problems, we employ random forest classifier which has been the preferred method for PA assessment using accelerometer data in the previous works [8, 11].

As another step in PA assessment, we also pursue energy expenditure (MET value) estimation to evaluate the performance of the models. We use random forest regression method to estimate the energy expenditure required to perform a PA.

## Results

Table 2 shows a summary of standard wrist variables for the performed activities of daily living, their mean (SD) METs, and the labels as sedentary or locomotion PAs. In our experiments, we divided our data into training and test sets. We used the collected from 126 participants for our training set and kept the other 20 for the test set. We tuned our machine learning methods using the training set and applied the trained models on the test set and reported their outcomes.

For activity identification, we used feature vectors for 15-second epochs to train the classifier. The classifier predicted labels for epochs of the same size for the test set data. For each activity in the test set, the label assigned to the majority of its epochs was selected as the predicted label. To estimate the energy expenditures for each activity, we used an average feature vector over all 15-second epochs for both training the model and estimation.

**Fig. 5:**
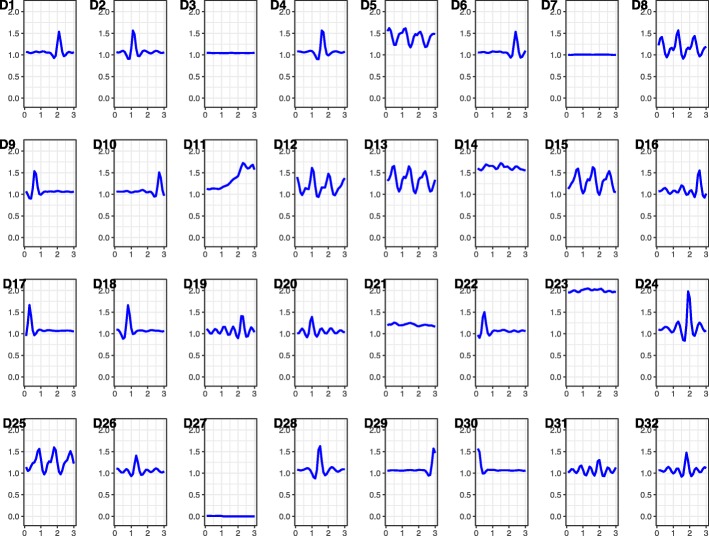
The 32-atom codebook learned for subsequence size of (*k*=) 3 s. Each of the atoms was the centroid and representative of a group of 3-second vector magnitude acceleration data in units of gravity – g (m/s^2^)

For the bag-of-words variable representations, we tried a variety of combinations for subsequence length (*k*∈{3 and6} s) and codebook size (*d*∈{8,16, 32, and64} words). The best performance was obtained using *k*=3 s for subsequent length and *d*=32 words for the codebook, and thus, we report the results using this parameter setting. Figure 5 shows the learned codebook with thirty-two 3-second words.

For sedentary PAs, D3 and D7 words (acceleration patterns) had the highest values. These words were representatives of accelerometer patterns with negligible intensities. The other words had values close to 0, which shows they were rarely observed during sedentary PAs and they had no association with PAs belonging to the sedentary category. D8 and D19 had the highest values for the locomotion category, and thus, the top indicators of consistent movements, such as walking. Interestingly, for stair ascending PAs the dominant acceleration patterns were D9 and D10. The combination of these two words yield an acceleration pattern resembling D8 but at a lower pace. This is due to the fact that ambulation in climbing stairs is slower than walks.

**Fig. 6:**
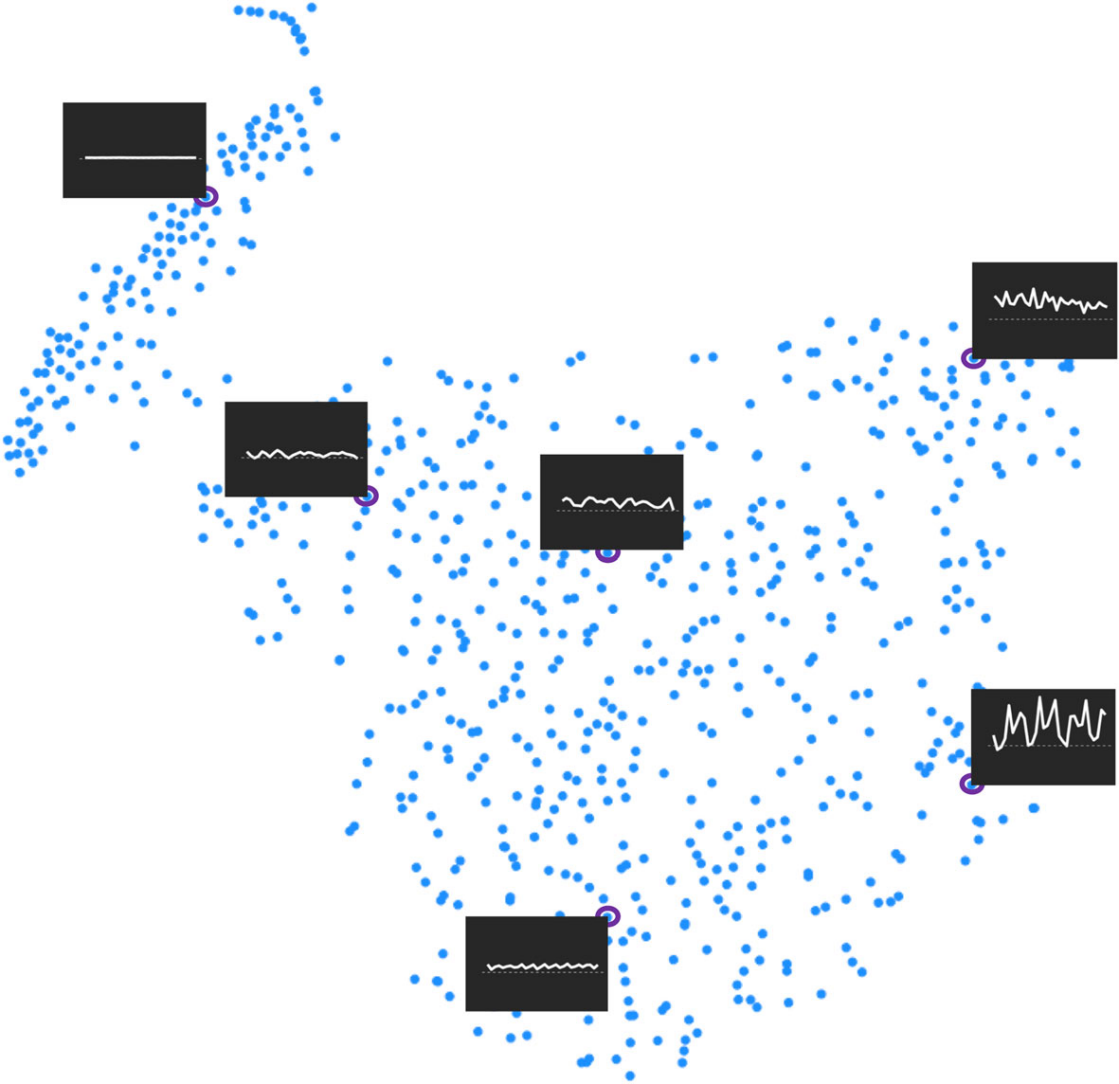
A two dimensional visualization of the codebook learned for locomotion activity with 3-second atoms. The distance between atoms are obtained from Dynamic Time Warping method. To preserve the distances for visualization, t-distributed stochastic neighbor embedding (t-SNE) was employed. The plotted atoms (dark background) were the medoids and representative of their clusters and selected for the final codebook

We used different atom lengths (*k*∈{3,6} s) and selection rates (*β*∈[0.01,0.1]) to learn the codebook using the supervised shape feature approach. Figure 6 shows the selected atoms for locomotion activities for atom length of 3 s and selection rate of 0.01.

We categorized activities of daily living into two classification problems: 1) sedentary versus nonsedentary and 2) locomotion versus stationary activities. Table 3 shows the performance of explained methods for sedentary-nonsedentary PA identification on the test set. Since the number of sedentary PAs were only a small fraction of the total number activities (9%), for a better assessment, we report accuracy, sensitivity, precision, and F1-score measures, which are calculated as follows:

$$\text{sensitivity} = \frac{\text{\# True Positives}}{\text{\# Positives}}{,} $$

$$\text{precision} = \frac{\text{\# True Positives}}{\text{\# Predicted Positives}}{.} $$

**Table 3:** Sedentary PA identification

	Accuracy	Sensitivity	Precision	F1-score
Standard Wrist	0.93	0.93	0.86	0.89
Bag-of-Words	0.97	0.92	0.80	0.85
SSF ^*a*^	0.96	0.88	0.85	0.86

^a^Supervised shape feature

F1-score is the harmonic mean of sensitivity and precision. For sedentary-nonsedentary classification problem, sedentary PAs are considered as *positive* samples.

Similar to the previous case, for locomotion-stationary classification problem, the number of samples belonging to each class was not evenly distributed (19% of the PAs belonged to locomotion class). Therefore, we use the above-mentioned evaluation metrics to compare the methods. The results are presented in Table 4.

**Table 4:** Locomotion PA identification

	Accuracy	Sensitivity	Precision	F1-score
Standard Wrist	0.92	0.82	0.92	0.87
Bag-of-Words	0.94	0.69	0.99	0.81
SSF ^*a*^	0.94	0.76	0.92	0.83

^a^Supervised shape feature

**Fig. 7:**
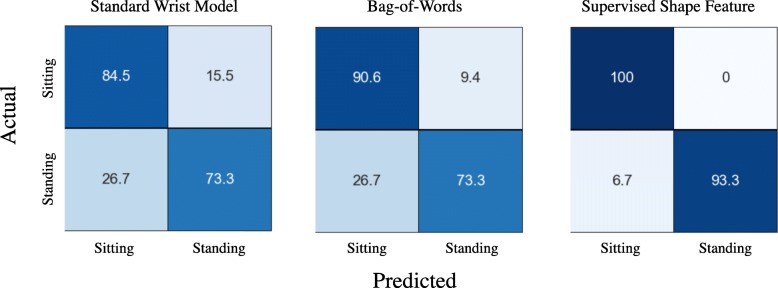
Confusion matrices for sedentary PAs obtained from standard wrist model, bag-of-words method, and supervised shape features. Each row represents actual PAs and columns show predicted labels

**Fig. 8:**
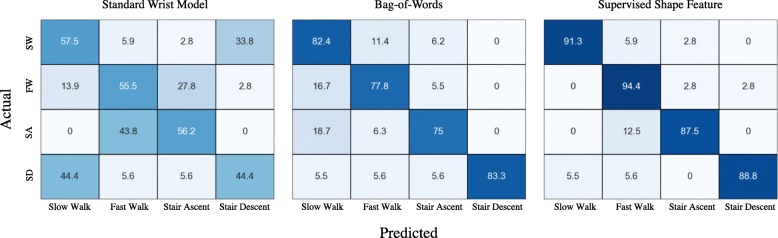
Confusion matrices for locomotion PAs obtained from standard wrist model, bag-of-words method, and supervised shape features. Each row represents actual PAs and columns show predicted labels. SW: Slow Walk; FW: Fast Walk; SA: Stair Ascent; SD: Stair Descent

Three PAs in our data were categorized as sedentary tasks; computer work, TV watching (i.e., sitting), and standing still. Figure 7 shows the confusion matrices obtained from each method. The supervised shape feature method provided the best performance for specific activity type identification (accuracy = 96.6%) compared with the standard wrist model (accuracy = 78.9%) and bag-of-words method (accuracy = 81.9%). We also obtained confusion matrices for PAs belonging to locomotion category. We considered Leisure Walk and Walk at RPE 1 as slow walks, and Rapid Walk and Walk at RPE 5 as fast walks. Figure 8 shows performance of each method.

We also pursued energy expenditure estimation as another part of our evaluations. We report the root mean squared error (rMSE) and the goodness of fit (R^2^) metrics to compare the performance of methods in estimating the energy expenditure required to perform activities of daily living. First, we trained models using the derived features to estimate the energy expenditures. Next, we included the predicted labels obtained from the sedentary and locomotion classifiers as two additional independent variables for regressors. Finally, we added the predicted labels for the specific activity type as 8 additional binary variables (2 sedentary and 4 locomotion PAs) to the energy expenditure estimation models. Table 5 shows rMSE and R^2^ values for energy expenditure (MET value) estimations. Overall, all methods performed similarly well and with low error. The standard wrist model outperformed the two shape feature representations when the original feature vectors were only considered; however, including the additional PA identification steps resulted in less noticeable improvement.

**Table 5:** Energy expenditure (MET value) estimations

	rMSE	Adjusted R^2^
SWM ^*a*^	0.9172	0.5178
SWM + Sedentary & Locomotion	0.9135	0.5197
SWM + Sedentary & Locomotion + Activity Type	0.9038	0.5275
BoW ^*b*^	1.0018	0.4241
BoW + Sedentary & Locomotion	0.9736	0.4573
BoW + Sedentary & Locomotion + Activity Type	0.9302	0.4992
SFF ^*c*^	0.9386	0.4911
SFF + Sedentary & Locomotion	0.9255	0.4992
SFF + Sedentary & Locomotion + Activity Type	0.8973	0.5380

^a^Standard wrist model

^b^Bag-of-Words approach

^c^Supervised Shape Feature

**Fig. 9:**
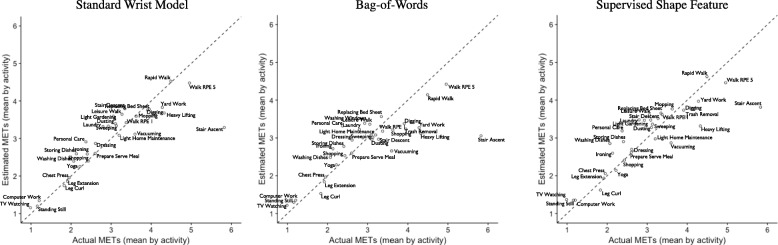
Mean performance of standard wrist, Bag-of-Words, and CovNet models to estimate average energy expenditure per PA

Figure 9 shows the average energy expenditure (MET) estimation for standard wrist model, Bag-of-Words approach, and supervised shape features. Stair ascent, as the only labor-intensive PA, was the most challenging activity for the methods to estimate the required energy expenditure.

## Discussion

In this study, introduced two shape feature derivation methods, which rely on supervised and unsupervised methods to learn codebooks. We compared the performance of the two new models with the standard wrist accelerometer model for PA recognition and energy expenditure estimation. While the standard wrist model outperforms the other feature representation for locomotion and sedentary PA identification (Tables 3 and 4), the shape feature methods are not far behind. For new target variables, e.g., identifying the specific activity type within sedentary or locomotion PAs, they are able to perform with modestly higher accuracies.

The standard wrist model offers high interpretability since the features are manually crafted. The shape feature methods also provide some level of interpretability; since these methods rely on the learned codebook, by checking the characteristics of the atoms and their weights for each PA insight could be obtained.

By definition, the energy expenditure (MET value) required to perform sedentary PAs is <1.6. Therefore, there is little variation in energy expenditure within this category. Table 2 shows that most of locomotion PAs (e.g., fast walks and stair ascent) require more energy than other activities of daily living, while the rest rank somewhere in the middle. Therefore, one might wonder whether it is possible to improve energy expenditure estimation by identifying the PA type first and training activity-specific energy expenditure models. For smaller datasets (<25 participants), previous works showed that this approach enhances energy expenditure models significantly [11, 23]. The standard wrist model is not able to identify the specific activity types within the locomotion class; for most of the cases, the model misclassifies slow walks as stair descents (or vice versa) and rapid walks as stair ascent (or vice versa). Since the MET ranges for the mentioned locomotion PAs are almost similar, the higher error rate for this model does not prevent it from benefiting from the additional steps. Although including further PA identification steps improves the energy expenditure estimation models, the improvements are not statistically significant. This is due to the fact that we obtain a model with lower bias if sufficient training data is provided. Thus, extra steps to reduce model’s error, which was a result of lack of data, are no longer necessary.

Besides the strengths, there are also limitations to this study. Although it compared three PA assessment models using wrist accelerometer data, it did not cover activity-count-based methods [24]. Another limitation is that the PAs were performed in laboratory settings. It has been shown that machine learning methods trained on such data perform poorly in free-living condition [25]. Therefore, future studies should consider including accelerometer data for activities of daily living in real-life conditions. This study employed participant-independent models for PA assessment. However, it has been shown that personalized models perform activity recognition with higher accuracy [26] and are subject to future works.

## Conclusion

The current study compared three different approaches for PA type identification and energy expenditure estimation using high-resolution accelerometer data collected on the wrist. The two shape feature derivation methods result in comparable performance for PA assessment. The standard wrist model provides interpretable features and is well suited for the sedentary and locomotion PA identification. However, it might not be adequate for other PA type identifications; e.g., detecting tasks which require upper/lower body movements. Therefore, depending on the PA type classification careful feature engineering ought to be pursued. Shape feature derivation methods provide feature vectors with less efforts. This is more apparent for the bag-of-words approach, which learns a codebook from the accelerometer data, regardless of the activities of interest. Thus, upon any alteration in the target variable (i.e., physical activity), the weights can be adjusted by re-applying the *tf*−*idf* method. Thus, when there is a variation in the PA assessment data analysis pipeline (e.g., new target variable) the introduced methods could be practical choices for feature derivation step.
